# Decitabine of Reduced Dosage in Chinese Patients with Myelodysplastic Syndrome: A Retrospective Analysis

**DOI:** 10.1371/journal.pone.0095473

**Published:** 2014-04-18

**Authors:** Xiao Li, Qiang Song, Yu Chen, Chunkang Chang, Dong Wu, Lingyun Wu, Jiying Su, Xi Zhang, Liyu Zhou, Luxi Song, Zheng Zhang, Feng Xu, Ming Hou

**Affiliations:** 1 Department of Hematology, the Sixth People’s Hospital affiliated with Shanghai Jiaotong University, Shanghai, China; 2 Department of Hematology, Qilu Hospital affiliated with Shandong University, Jinan, China; 3 Department of Hematology, Ruijin Hospital affiliated with Shanghai Jiaotong University School of Medicine, Shanghai, China; Queen’s University Belfast, United Kingdom

## Abstract

Decitabine has been approved for the treatment of all subtypes of myelodysplastic syndrome (MDS). However, the optimal regimen for decitabine treatment is not well established. In this study, an observational, retrospective and multi-center analysis was performed to explore the decitabine schedule for the treatment of MDS. A total of 79 patients received reduced dosage decitabine treatment (15 mg/M^2^/day intravenously for five consecutive days every four weeks). Fifty-three out of the 79 patients were defined as intermediate-2/high risk by international prognostic scoring system (IPSS) risk category. 67.1% of MDS patients achieved treatment response including complete response (CR) (n = 23), Partial response (n = 1), marrow CR (mCR) with hematological improvement (HI) (n = 11), mCR without HI (n = 11) and HI alone (n = 7) with a median of 4 courses (range 1–11). The median overall survival (OS) was 18.0 months. The median OS was 22.0, 17.0 and 12.0 months in the patients with CR, those with other response, and those without response, respectively. In addition, this regimen contributed to zero therapy-related death and punctual course delivery, although III or IV grade of cytopenia was frequently observed. In conclusion, the 15 mg/M^2^/d×5 day decitabine regimen was effective and safe for Chinese MDS patients with IPSS score of 0.5 or higher.

## Introduction

Myelodysplastic syndrome (MDS) is widely recognized as a clonal hematopoietic stem cell disorder. The hypermethylation of tumor suppressor genes (TSGs) is frequently observed in MDS, which may play a key role in the pathogenesis of MDS [1], [2]. Decitabine has been approved for the treatment of MDS of all FAB subtypes and different International Prognostic Scoring System (IPSS) risk groups [3]. The use of decitabine is often limited by its severe toxicity represented by myelosuppression even at relatively low doses [4]–[6].

Reported number of MDS patients receiving decitabine treatment is still limited at less than 600 cases [7]–[14]. As a result, ideal regimen is not known [15]. A low-dose 5-day decitabine regimen (20 mg/M^2^/d for 5 days every four weeks) is widely used in many oncology centers worldwide and seems to have better efficacy and safety profile in comparison to a high dose 3-day protocol (for a total of 135 mg/M^2^ per course) [7], [8]. However, treatment-related death is still estimated to occur in 6% of the patients receiving this extended regimen at low dose if the dose and schedule is not adjusted properly [10]. To minimize the risk of myelosuppression and death, treatment must often be postponed at the cost of efficacy [9], [11].

In a dosage-exploration trial of small scale [5], reduced decitabine at 15 mg/M^2^/d for 10 days (150 mg/M^2^ per course) achieved very promising response. Due to severe hematological adverse events, however, the trial was terminated. In a mechanistic study by Yang *et al*. [6] in patients with hematologic malignancies, decitabine at 15 mg/M^2^/d produced comparable degree of hypomethylation of the Alu and LINE1 elements in comparison to 20 mg/M^2^/d. This study also showed that low doses at 15 and 20 mg/M^2^/d have more efficacious hypomethylation action than at a daily dose of 100 mg/M^2^, which seemed to mainly drive a cytotoxic effect. Put together, these findings seem to advocate a daily dose of 15 mg/M^2^ but for less duration (than 10 days in a course).

In the current study, we carried out a preliminary assessment of a decitabine regimen at 15 mg/M^2^/d for 5 days per course in Chinese MDS patients with IPSS score of 0.5 or higher.

## Methods

### Patients

This multi-center, retrospective study was approved by the institutional Review Board of all participating centers. All subjects signed written informed consent. Disease subtype was classified based on the French-American-British (FAB) classification [16]. The IPSS [17] score was 0.5 or higher. Patients having an Eastern Cooperative Oncology Group performance score of >2, receiving previous hypomethylation therapy, or co-morbid with severe heart-lung diseases were excluded from data analysis.

### Ethics Statement

All subjects provided written informed consent between September 2009 and June 2013 from three Chinese hematological institutes. The written informed consent was obtained from patient themselves (blank copy of informed consent in Table S1). The study was approved by the ethics committee of the Sixth People’s Hospital affiliated with Shanghai Jiaotong University, Qilu Hospital affiliated with Shandong University and Ruijin Hospital affiliated with Shanghai Jiaotong University School of Medicine. All patient-relevant research strictly abided by the Declaration of Helsinki.

### Treatment

Decitabine was infused intravenously over a one-hour period at a daily dose of 15 mg/M^2^ for five consecutive days every four weeks. Neither dose reduction nor escalation was allowed, but the treatment course was delayed upon grade 4 hematologic toxicities or life-threatening myelosuppression (e.g., bleeding or proven infection). The patients accepted decitabine treatment for at least four courses unless the disease progressed, or the patients experienced intolerable myelosuppression. Prophylactic antimicrobials, hematopoietic growth factors, and other supportive cares were available at the discretion of the physician.

### Response and Hematological Toxicity Evaluation

A routine blood examination was performed twice every week. Bone marrow (BM) was examined with routine aspirate smear and G-banding analysis every 1–2 treatment courses to evaluate responses. The primary endpoint was overall response rate (ORR) using the IWG 2006 criteria [18], and included complete response (CR), partial response (PR), marrow CR (mCR), hematological improvement (HI) and cytogenetic response (including cytogenetic CR (cCR) defined as no detectable cytogenetic abnormality and cPR defined as at least 50% reduction in abnormal metaphases). The secondary endpoints included overall survival (OS), and the rate of acute myeloid leukemia (AML)-free survival at one year from the beginning of treatment [9]–[11].

Hematological side-effects were assessed using Common Toxicity Criteria version 3.0 [19], including cytopenia, proven infections. Death within 30 days from the beginning of treatment was also calculated.

### The p15^INK4B^ Methylation Status

The methylation status of p15^INK4B^ was examined in BM mononuclear cells with a methylation-specific PCR (MSP) method, as previously described [20].

### Statistical Analysis

The χ2 test were used to compare categorical variables. Time-to-event was presented using the Kaplan-Meier method and analyzed with a log rank test. All statistical analyses were carried out using the SPSS 16.0 statistical software.

## Results

### Patient Demographics

A total of seventy-nine patients were treated by decitabine between September 2009 and June 2013 by three Chinese hematological institutes. The median age was 60 years old (range 28–82 years), and the male-to-female ratio was 49∶30. The characteristics of these patients were detailed in Table 1. Based on the IPSS risk category, 53 patients had higher-risk MDS (Int-2 and high risk) and the other 26 had lower-risk (Int-1 risk), respectively. Forty-four patients (55.7%) had abnormal karyotypes prior to decitabine treatment, with twenty (25.3%) patients having poor-risk karyotypes based on the IPSS risk category. The median number of decitabine courses was 4 (range 1–11).

**Table 1:** Clinical characteristic of MDS patients.

	Values
Regimen	15 mg/M^2^/d ×5
Tested patients, n	79
Median age, y	60 (28–82)
M:F ratio, n	49∶30
De novo MDS, n (%)	74 (93.7)
RAEB plus RAEB-t, n (%)	59 (74.7)
CMML, n (%)	7 (8.9)
IPSS int-1 risk, n (%)	26 (32.9)
IPSS int-2/high risk, n (%)	53 (67.1)
Abnormal chromosome, n (%) according to IPSS	44 (55.7)
Good, n (%)	40 (50.6)
Intermediate, n (%)	19 (24.1)
Poor, n (%)	20 (25.3)
p15^INK4B^ positive, n (%)	40/59 (67.8)

Abbreviations: RAEB, refractory anemia with excess blasts; RAEB-t, refractory anemia with excess blasts in transformation; CMML, chronic myelomonocytic leukemia; IPSS, International Prognostic Scoring System.

### Treatment Response

Fifty-three out of the 79 patients (67.1%) responded to the treatment (Table 2): CR in 23 cases (29.1%), PR in one case (1.3%), mCR without HI in 11 cases (13.9%), mCR with HI in 11 cases (13.9%), and HI alone in 7 cases (8.9%). Cytogenetic response was evaluable in 37 patients, and included 17 (45.9%) CR, 4 (10.8%) PR and 16 (43.2%) NR.

**Table 2:** Response data (79 patients) by the 2006 IWG Criteria.

Response by IWG 2006 criteria	Patients number (%)
ORR	53 (67.1)
CR	23 (29.1)
PR	1 (1.3)
mCR without HI	11 (13.9)
mCR with HI	11 (13.9)
HI only	7 (8.9)
SD	5 (6.3)
PD	21 (26.6)
Cytogenetic response* n (%)	
CR	17/37 (45.9)
PR	4/37 (10.8)
NR	16/37 (43.2)

*the percentage means the ratio of responder in 37 patients with abnormal karyotypes.

Abbreviations: IWG, International Working Group; ORR, overall response rate; CR, complete response; mCR, marrow CR; PR, partial response; HI, hematologic improvement; SD, stable disease; PD, progressive disease.

### Relationship between Treatment Response and p15 Methylation Level

**Figure 1:**
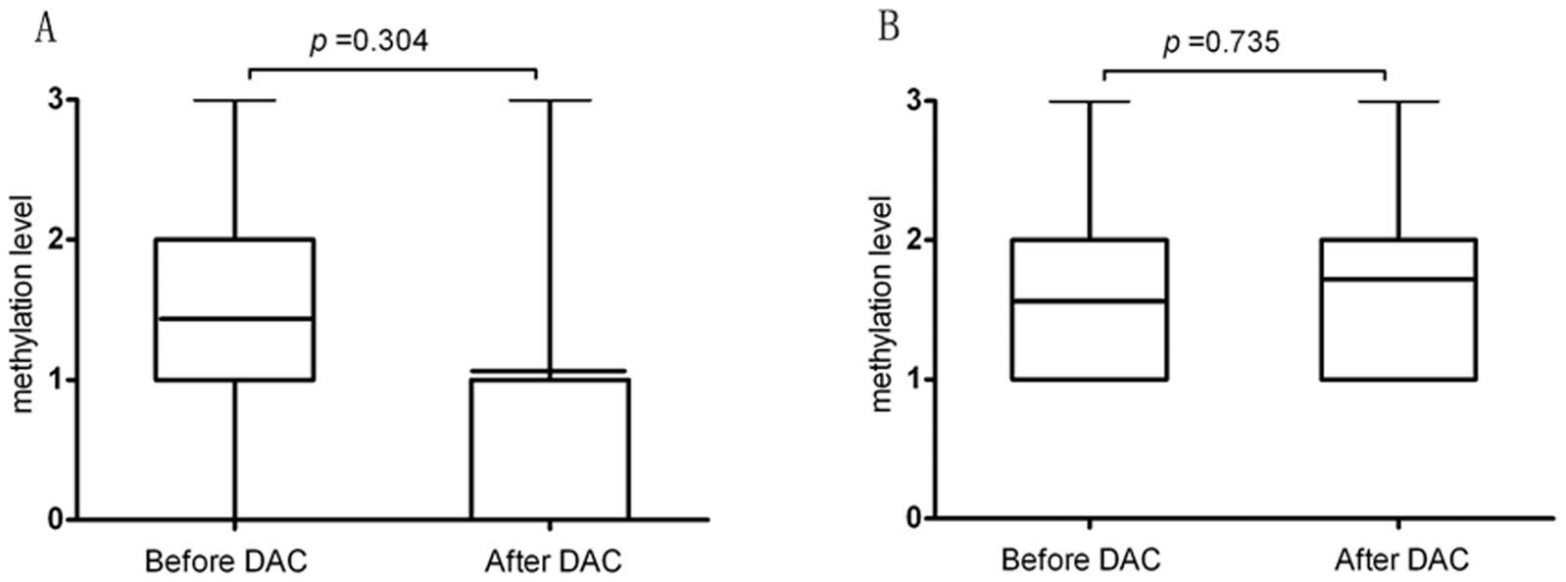
Comparison of P15^ink4b^ methylation before and after decitabine regimens. p15 methylation level decreased after decitabine treatment in responders (n = 41) (A); p15 methylation level elevated slightly after decitabine treatment in nonresponders (n = 18) (B).

The results (detected by semi-quantitative MSP-PCR) are illustrated from low to high, according to the levels of DNA methylation (M) or unmethylation (U), and were classified as negative (no visible M band, represented by grade 0), weakly positive (M weaker than U, represented by grade 1), positive (M equal to U, represented by grade 2), or strongly positive (M stronger than U, represented by grade 3). Neither ORR nor CR differed between the patients with p15^INK4B^ methylation (ORR 61.7% and CR 23.4%;) vs. without.(ORR 65.6% and CR 28.1%;). Although the P15 methylation level declined for those responders after decitabine treatment, the change did not appear statistically significant. (see Figure 1).

### Hematological Toxicity

Major side-effects included cytopenia and cytopenia-related infection (Table 3). Grade III or IV neutropenia (70.9%), and thrombocytopenia (67.1%) were frequently observed. The zero of neutrophil count and thrombocytopenia less than 10×10^9^/L occurred in 7.6% and 24.1% of patients, respectively. Non-hematological toxicities were infrequent and reversible. None of the patients died within 30 days of the initiation of decitabine treatment. In addition, although 32 of 79 (40.5%) patients had 58 courses delayed due to grade 4 hematologic toxicities or life-threatening myelosuppression in this study. 63% of the decitabine courses (156/214 courses not including the first 79 courses for the 79 patients) were delivered on schedule, with a median interval between the courses at 28 days (range 28–61 days).

**Table 3:** Hematological adverse effects*.

	Number of patients (%)
Neu III*	13 (16.5)
Neu IV*	43 (54.4)
Neu = 0	6 (7.6)
PLT III	7 (8.9)
PLT VI	46 (58.2)
PLT ≤10×109/L	19 (24.1)
Proven infection	18 (22.8)
Death within 30 days#	0

*The grading was based on the Common Toxicity Criteria for Adverse Events version 3.0.

# within 30 days of the start of the initial course of decitabine.

### Survival Data

**Figure 2:**
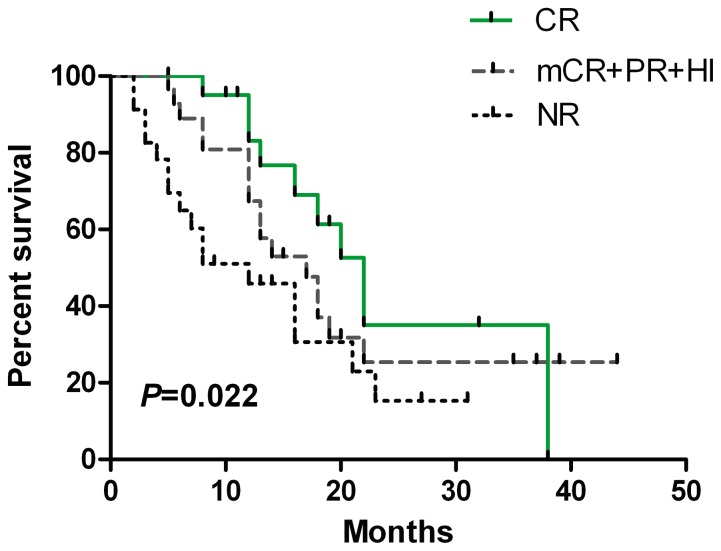
Overall survival according to treatment response type. The overall survival (OS) of the patients who reached CR; or achieved the other response (PR or mCR with/without HI); or got no any response after decitabine treatment; had different OS (22.0 vs. 17.0 vs. 12.0 months, respectively, *P* = 0.022). The patients with CR had longer survival than those without treatment response (22.0 vs. 12.0 months, *P* = 0.001).

**Figure 3:**
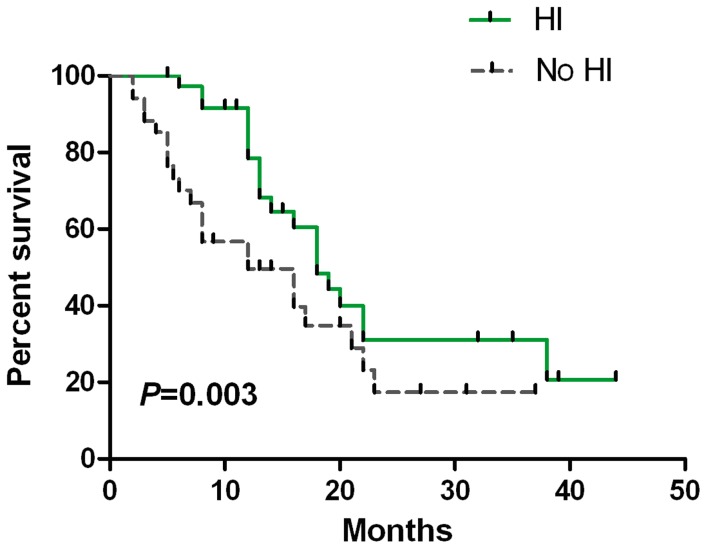
Overall survival according to different patients who achieved HI or did not achieved HI. Patients with HI (CR, PR, mCR with HI or Hi alone) also showed longer OS (18.0 months) than those without HI (mCR alone, failure or PD). (12 months, *P* = 0.003).

Median OS was 18.0 months (95% CI, 14.7–21.3 months). The rate of OS and AML-free survival at one year was 63.3% and 60.8%, respectively. The median OS was 22.0, 17.0 and 12.0 months in the patients with CR, in those with other types of response (PR or mCR with/without HI), and in those without response to decitabine, respectively (*P* = 0.022). Obvious significance could be observed between the patients with CR and those with NR (*P = *0.001) (Figure 2). The OS was also longer in the patients with HI (CR, PR, mCR with HI or HI alone) than in those without HI (mCR alone, failure or PD) (18.0 vs. 12.0 months, *P* = 0.003) (Figure 3).

### Comparison of Current Study with Previous Reports

We compared our results with three previously reported data using 5-day regimen (20 mg/M^2^/d or 100 mg/M^2^/course, Table 4) [9]–[11]. Our results were similar to the MD. Anderson study [9] on ORR (67.1% vs. 71.0) and CR (29.1% vs. 33.7%). The OS from current study was comparable with data from the other three studies. And our results showed punctual median course interval of 28 days without therapy-related death.

**Table 4:** Comparison of current study with previous reports.

	Current study	ID-03-0180 MD.Anderson	Daco-020 ADOPT	DIVA study
No. patients	79	95	99	101
Median age, years	60 (28–82)	65 (NM*)	72 (34–87)	65 (23–80)
Eligibility	FAB MDS (IPSS ≥0.5)	FAB MDS (IPSS ≥0.5)	FAB MDS (IPSS ≥0.5)	WHO (IPSS ≥0.5)+CMML
De novo MDS (%)	94	68	89	89
IPSS ≤1.0	32.9%	34.0%	54.0%	52.0%
≥1.5	67.1%	66.0%	46.0%	48.0%
Decitabine regimen	15 mg/M^2^/d×5 d	100 mg/M^2^/course (3 schedules)	20 mg/M^2^/d×5 d	20 mg/M^2^/d×5 d
courses, median (range)	4 (1–11)	>7 (1–18)	5 (1–17)	5 (1–18)
Treatment response				
CR	29.1%	33.7%	17.0%	12.9%
PR	1.3%	1.0%	0	1.0%
mCR	27.8%	25.0%	15.0	22.8%
HI	8.9%	13.0%	18.0%	18.8%
ORR	67.1%	71.0%	51.0%	55.4%
Overall survival				
Median	18.0 months	19.0 months	19.4 months	17.7 months
1-year probability	63.3%	56.0%	66.0%	78.6%
1-year % for AML-free survival	60.8%	51.%∞	NM	77.9%
Median Course interval	28 day	35–40 days	28 days	34 days
Death in 30 days (n)	0	0	11^#^	NM*

11^#^ refer to reference 10. just 6/11 patients death was considered being related to decitabine treatment. NM* means not mentioned; ^∞^this datum was from a observation at the point of 18 months.

## Discussion

The hypermethylation of tumor suppressor genes (TSGs) plays a key role in the pathogenesis of MDS [1], [2]. Decitabine reduces DNA methylation, which in turn promotes re-expression of TSGs, and by doing so, inhibits tumor growth [4]–[6]. Despite of the apparent efficacy, the three-day protocol (135 mg/M^2^/course every 6 week) of decitabine lead to high therapy-related death [7], [8]. Dose reduction (five-day regimen at 100 mg/M^2^/course every four week) reduces therapy-related death to approximately 6%, a level still not satisfying [10]. To minimize life-threatening toxicity, scheduled course often needs to be delayed [9], [11]. A pilot dose-finding study indicated that 15 mg/M^2^/d is optimal. Similar degree of demethylation was achieved at 15 mg/M^2^/d in comparison to 20 mg/M^2^/d on Alu and LINE1 elements [4], [5]. All together, these information suggested feasibility of the five-day regimen of 15/M^2^/d decitabine (75 mg/M^2^/course every four weeks).

In this study, fifty-three (67.1%) of the 79 patients achieved treatment response, including 23 patients (29.1%) with CR and 21 patients (21/37, 56.8%) with cytogenetic response. The median OS for the patients receiving decitabine treatment reached 18 months, whereas the median OS in MDS patients receiving the best care in previous report was only 8.5 months [8]. These data suggested that the modified decitabine regimen (15 mg/M^2^/d for 5 days) is effective in MDS patients with an IPSS score at 0.5 or higher. Besides, 63% of course delivery could be punctually performed under this regimen (the median course interval was 28 days) without therapy-related death, although III or IV grade of cytopenia was frequently found in over 50% of patients. Our results suggested that this regimen was not only effective, but also relatively safe. At the same time, it was similar with previous report, that the methylation of p15^INK4B^ in this assay did not predict the decitabine response [4].

A comparison of the current 5-day regimen (75 mg/M^2^/course) with previous studies [9]–[11] showed similar ORR and CR as reported by Kantajian *et al.* [9], and slightly better than that in the Steensma *et al.* [10] and *Lee et al.* [11] studies. A recent study on decitabine treatment containing 66.7% of MDS patients with IPSS Int-1 risk reported only 22.4% ORR and 10.5% CR [21]. ORR and CR in the ADOPT and DIVA trials were relatively low because over 50% of patients in the studies had IPSS low or Int-1 risk.

None of the 79 patients died of decitabine treatment in our study. Also, 63% of the decitabine courses were delivered on schedule (median course interval at 28 days). The data from ADOPT trial [10] and Korea [11], reported a delay of scheduled decitabine treatment (median course interval at 35–40 and 34 days due to severe cytopenia, respectively). The ADOPT trial reported 11 deaths during the initial 30 days of decitabine treatment, with six out of the 11 deaths attributable to decitabine treatment. In other word, delay in schedule treatment and therapy-related death in the 20 mg/M^2^/d regimen may influence the therapeutic effect of decitabine. In comparison to the standard regimen of 20 mg/M^2^/d, a regimen with reduced dosage (15 mg/M^2^/d) could achieve similar clinical response but minimize course delay and none-therapy-related death. Although some prognostic factor analysis [22] did not refer to this probably important factor,we considered that the increased adherence to scheduled treatment may contribute to satisfying effects despite of reduced dosage.

Primarily, the reduced decitabine regimen (15 mg/M^2^/d for five consecutive day every four weeks) appeared effective and relatively safe in Chinese patients with MDS. But, because of the limitation of an observational and retrospective analysis, some big prospective and double-blind clinical trials are necessary to validate our result.

## Supporting Information

Table S1 **Informed Consent Form.**
(DOC)
