# Supplementary material for: Decitabine of Reduced Dosage in Chinese Patients with Myelodysplastic Syndrome: A Retrospective Analysis
Source: PLoS One. 2014 Apr 18;9(4):e95473. doi: 10.1371/journal.pone.0095473 (PMC3991661; doi:10.1371/journal.pone.0095473)
Supplement: Table S1 — Informed Consent Form. (DOC) [file pone.0095473.s001.doc]

**Table S1 Informed Consent Form**

Dear subjects:

Your medical records in XX hospital from (Day)/ (Month)/ (Year)/ to (Day)/

(Month)/ (Year)/ may be used in a clinical trial titled “A retrospective clinical trial on decitabine for myelodysplastic syndrome” if you permitted to do this. You should carefully observe the following information before making a decision to participate or not. If you cannot understand any of the following contents clearly, you can ask for the fully interpretation from the responsible physicians.

1. **Objectives**

This clinical trial was designed to evaluate the efficacy and safety of decitabine for myelodysplastic syndrome patients with IPSS score of 0.5 or higher.

1. **Confidentiality and original data review**

As the findings of this clinical trial is collected solely for the purpose of scientific research, the detailed information of your participation and private data generated due to the participation should be maintained confidentially and will be protected according to the local laws and regulations. Your name and identity will not be disclosed. Only the members of independent ethic committee and investigators have an access to your research data for the purpose of researching when they do this in accordance with the local regulations. During the publication of the data of this trial, your personal identity will not be disclosed.

1. **Risks resulting from the participation**

As this is a retrospective study, and no intervention should be performed for your therapeutic regimen, the participation into this trial will not increase your risk for the treatment of the disease.

1. **Voluntariness**

You should completely voluntary to participate into this study as your refusal will not have influence on the treatment and attitude taken by the investigators.

**Patient or legal representative statements**

1. After sufficient consideration, I am willing to participate into the trial titled “A retrospective clinical trial on decitabine for myelodysplastic syndrome”.
2. I agree to collect, use and publish my medicine-related data in this medical trial.

Signature (subject): Tel.:

Date:

Signature (physician): Tel.:

Date:
